# Feasibility of Diffusion Tensor and Morphologic Imaging of Peripheral Nerves at Ultra-High Field Strength

**DOI:** 10.1097/RLI.0000000000000492

**Published:** 2018-11-07

**Authors:** Annina B. Schmid, Jon Campbell, Samuel A. Hurley, Saad Jbabdi, Jesper L. Andersson, Mark Jenkinson, Neal K. Bangerter, David L. Bennett, Irene Tracey, Robert Frost, Stuart Clare

**Affiliations:** From the * Nuffield Department of Clinical Neurosciences,; † Wellcome Centre for Integrative Neuroimaging, Nuffield Department of Clinical Neurosciences, Oxford University, Oxford, United Kingdom;; ‡ School of Medicine and Public Health, University of Wisconsin-Madison, Madison, WI;; § Department of Bioengineering, Imperial College London, UK;; ∥ Athinoula A. Martinos Center for Biomedical Imaging, Massachusetts General Hospital, Charlestown; and; ¶ Department of Radiology, Harvard Medical School, Boston, MA.

**Keywords:** diffusion tensor imaging, 7 T, structural imaging, peripheral nerve, carpal tunnel syndrome, peripheral neuropathy, fractional anisotropy, radial diffusivity, ultra-high field strength, magnetic resonance imaging

## Abstract

**Objectives:**

The aim of this study was to describe the development of morphologic and diffusion tensor imaging sequences of peripheral nerves at 7 T, using carpal tunnel syndrome (CTS) as a model system of focal nerve injury.

**Materials and Methods:**

Morphologic images were acquired at 7 T using a balanced steady-state free precession sequence. Diffusion tensor imaging was performed using single-shot echo-planar imaging and readout-segmented echo-planar imaging sequences. Different acquisition and postprocessing methods were compared to describe the optimal analysis pipeline. Magnetic resonance imaging parameters including cross-sectional areas, signal intensity, fractional anisotropy (FA), as well as mean, axial, and radial diffusivity were compared between patients with CTS (n = 8) and healthy controls (n = 6) using analyses of covariance corrected for age (significance set at *P* < 0.05). Pearson correlations with Bonferroni correction were used to determine association of magnetic resonance imaging parameters with clinical measures (significance set at *P* < 0.01).

**Results:**

The 7 T acquisitions with high in-plane resolution (0.2 × 0.2mm) afforded detailed morphologic resolution of peripheral nerve fascicles. For diffusion tensor imaging, single-shot echo-planar imaging was more efficient than readout-segmented echo-planar imaging in terms of signal-to-noise ratio per unit scan time. Distortion artifacts were pronounced, but could be corrected during postprocessing. Registration of FA maps to the morphologic images was successful. The developed imaging and analysis pipeline identified lower median nerve FA (pisiform bone, 0.37 [SD 0.10]) and higher radial diffusivity (1.08 [0.20]) in patients with CTS compared with healthy controls (0.53 [0.06] and 0.78 [0.11], respectively, *P* < 0.047). Fractional anisotropy and radial diffusivity strongly correlated with patients' symptoms (*r* = −0.866 and 0.866, respectively, *P* = 0.005).

**Conclusions:**

Our data demonstrate the feasibility of morphologic and diffusion peripheral nerve imaging at 7 T. Fractional anisotropy and radial diffusivity were found to be correlates of symptom severity.

## Abstract 2

Supplemental digital content is available in the text.

Peripheral nerve imaging, also referred to as magnetic resonance neurography,^1^ represents a growing area in magnetic resonance imaging (MRI).^2^ Most methodologies have concentrated on enhancing neural signal in morphologic sequences. Over the past decade, diffusion tensor imaging (DTI) and its ability to provide information about neural microstructure has gained increasing interest in the field of peripheral nerve imaging.^3^ However, MRI of peripheral nerves remains challenging mostly due to the thin nature of these structures. Technical advances and particularly the availability of higher field strength have significantly improved visualization of peripheral nerves. Recently, morphologic peripheral nerve imaging has been performed at 7 T,^4–7^ which substantially enhances the signal-to-noise ratio (SNR) and therefore the achievable spatial resolution, thus improving anatomical depiction of peripheral nerves. In contrast to morphologic sequences, most human peripheral nerve DTI has been implemented at lower field strength, with the exception of one recent attempt at 7 T in 3 healthy participants.^4^ Studies performed in the central nervous system suggest that imaging at 7 T may improve diagnostic accuracy to detect neural pathology,^8^ something that bears even more importance when imaging comparably thin peripheral nerves.

The purpose of this study is therefore to describe the development, strengths, and limitations of morphologic and DTI sequences of peripheral nerves at 7 T, using carpal tunnel syndrome (CTS) as a model system of focal nerve injury. Correlations of MRI parameters with electrodiagnostic parameters and symptom severity will be established.

## MATERIALS AND METHODS

This prospective study (December 2014 to December 2016) was approved by the national ethics committee, and all participants gave written informed consent. Magnetic resonance imaging was performed on a 7 T whole-body MR system (MAGNETOM; Siemens Healthcare, Erlangen, Germany, 70 mT/m) with a dedicated 16-channel transmit-receive wrist array-coil (RAPID Biomedical, Germany, both devices are investigational). Six healthy participants volunteered for wrist scanning (3 females; mean age, 30.3 years [SD 7.11]). Eight patients with electrodiagnostically confirmed CTS^9^ were recruited from hand surgery departments (4 females; mean age, 55.3 years [SD 11.5]). Participants with evidence of neuropathies other than CTS (eg, ulnar neuropathy, cervical radiculopathy) or with MRI contraindications were excluded. Participants were positioned prone with the scanned hand above their head (“superman” position). This position optimally places the wrist in the isocenter of the magnet, but is often associated with discomfort. We therefore limited the acquisition time to a maximum of 30 minutes, which was tolerated by all participants.

### Clinical Parameters

Standard electrodiagnostic tests were performed as previously described,^10^ including median sensory and motor amplitudes and conduction velocities. Patients' symptom severity was established with the Boston symptom scale (see Appendix Table 1, Supplemental Digital Content, http://links.lww.com/RLI/A392, for clinical severity of patients with CTS).

### Morphologic Scans

Three-dimensional balanced steady-state free precession (SSFP) images were acquired using a multiple-acquisition phase-cycled technique, where 2 constituent phase-cycled images (variation of phase of radiofrequency pulses) were combined using root sum of squares to mitigate the banding artifacts characteristic of balanced SSFP imaging.^11^ In-plane resolution was 0.2 × 0.2 mm, and slice thickness was 0.4 mm (see Table 1 for scan parameters).

**TABLE 1:**
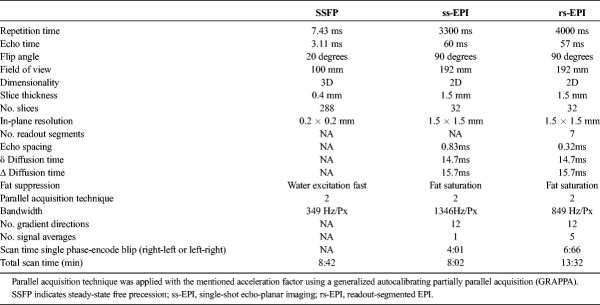
Scan Parameters

Nerve cross-sectional areas (CSAs) and normalized signal intensity (SI) of the median and ulnar nerves were quantified at the level of the radioulnar joint, pisiform, and hamate bone by manually outlining the nerves (by A.B.S.). Because of its division into small branches, the CSAs for the ulnar nerve were not determined at the hamate level. For SI, the average pixel intensity within the CSAs over 3 adjacent slices was divided by the average gray level of the pronator quadratus or hypothenar muscle.^12^

### Diffusion Imaging

#### Data Acquisition

We compared 2 methods of k-space sampling: single-shot echo-planar imaging (ss-EPI)^13^ and readout-segmented EPI (rs-EPI).^14^ Readout-segmented EPI has successfully been used at 3 T for imaging of the mandibular nerve with a 64-channel head-neck coil^15^ and has the advantage of shorter echo-spacing and echo train durations, thereby reducing susceptibility-induced distortion and T2* blurring.^16^ However, rs-EPI requires longer scan times than ss-EPI. To stay within tolerable scan times, the rs-EPI acquisition was limited to 1 repetition of 12 directions with 1 b = 0 s/mm^2^ image, whereas 5 repetitions of 12 directions with 5 b = 0 weighted images interleaved were acquired for ss-EPI. Diffusion weighting of b = 1000 s/mm^2^ was used for both sequences. Comparison of acquisitions with 5 × 12 directions versus 1 × 60 directions showed that 12 directions were sufficient to obtain comparable fractional anisotropy (FA) values (Appendix Figure 1, Supplemental Digital Content, http://links.lww.com/RLI/A392). The repetition of fewer directions increases the chances of obtaining at least 1 complete direction set should a scan be interrupted. The phase-encode direction was right-left and was reversed by 180 degrees for a second acquisition to allow for susceptibility-induced distortion correction during postprocessing. All scan parameters are summarized in Table 1.

#### DTI Postprocessing

Data were postprocessed using FMRIB's Software Library,^17^ which is a freely available library of MRI analysis tools. Previous work has shown that different software packages provide modest to substantial intratester and intertester reliability when analyzing DTI data.^18^ From the 2 acquisitions with opposing phase encode blips, which are distorted in opposite directions along the phase-encoding axis, the susceptibility-induced off-resonance field was estimated using the TOPUP tool^19^ as implemented in FMRIB's Software Library.^20^ Subsequently, the EDDY tool was applied to correct for eddy-current–induced distortions and rigid-body movement.^21^ We quantified the correction of distortion artifacts as the mean correlations of reversed phase-encoded acquisitions at each slice in healthy participants before and after TOPUP. Signal-to-noise ratio and SNR efficiency 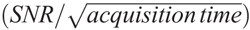 within the median nerve were determined for both ss-EPI and rs-EPI acquisitions from the first b = 0 image, using a difference method on the 2 distortion-corrected reversed phase encode images.^22^

We then used DTIFIT^21^ to create FA maps and FMRIB's Linear Image Registration Tool^23^ to register the diffusion images with the morphologic images (linear registration, 6 degrees of freedom, correlation ratio cost function). Registration success was visually judged (by A.B.S.) by overlapping the morphologic images and registered FA maps and grading the alignment of the median nerve in the 2 images at the level of the pisiform bone (perfect, partial, or no alignment). Estimates of median and ulnar nerve FA as well as median nerve mean diffusivity (MD), axial diffusivity (AD), and radial diffusivity (RD) were obtained using regions of interest (ROIs; average of 3 slices) at the 3 wrist levels. All quantification was performed by an investigator with 7 years experience in MRI research (A.B.S.). To determine intertester reliability, a second rater with less than 1 year of experience in MRI interpretation also performed the ROI analysis for all DTI measures in the middle of the carpal tunnel (pisiform bone).

### Statistical Analysis

Statistical analysis was performed with SPSS version 24 (IBM). The success of distortion correction was evaluated with paired *t* tests on the mean correlation between the reversed phase-encode images, before and after TOPUP correction.

Intertester reliability of DTI measurements was determined for all DTI measures using intraclass correlation coefficient (ICC, 3.2). As diffusion measures are age-dependent^24^ and our sample was not matched for age, we used analyses of covariance corrected for age to compare all MRI parameters between healthy participants and CTS patients (significance *P* < 0.05). For DTI, ROIs with signal loss were excluded from the analysis.

Pearson correlations with Bonferroni correction (5 comparisons: *P* < 0.01) were used to determine associations of MRI parameters in the middle of the tunnel (pisiform bone) with clinical parameters (Boston symptom score, median sensory nerve action potential amplitude and conduction velocity, median distal motor latency, and compound motor action potential amplitude) in patients with CTS.

## RESULTS

### 7 T Produces High-Resolution Morphologic Images

**FIGURE 1:**
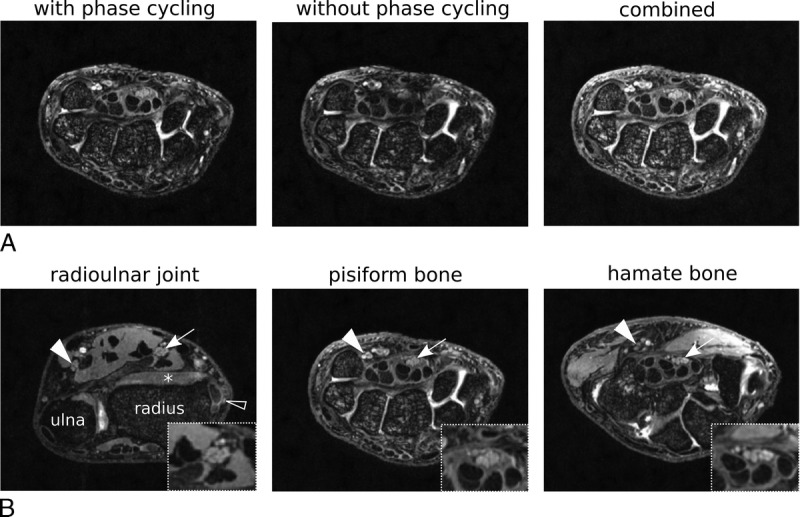
T2-weighted 3-dimensional balanced steady-state free precession (SSFP) images of the wrist acquired at 7 T. A, Reduction of banding artifacts in the SSFP images. The panels show transaxial SSFP images through the wrist acquired with and without phase cycling with clearly visible banding artifacts. The combined sum of square image largely eliminates banding artifacts, despite the severity in the two original images. B, Combined SSFP images. Panels show the median nerve (arrow) at the level of the radioulnar joint (left), pisiform bone (middle), and hook of hamate (right). The high spatial resolution of the 7 T images allows high visibility of morphologic details such as the differentiation of fascicles within the median nerve (zoomed insets). The 7 T morphologic sequences also allow visualization of the ulnar nerve (filled arrowhead) and the superficial radial nerve (empty arrowhead) at the wrist. The peripheral nerves are isointense with muscle (asterisk).

Figure 1 demonstrates the high in-plane resolution and banding artifact correction achievable with the morphologic 7 T sequences. Median and ulnar nerves were imaged with a high level of morphologic detail, with visualization of fascicles as well as clear boundaries of the nerves and tendons (Fig. 1B).

Quantification revealed that the CSA of the median nerve was larger in patients with CTS at the hamate bone (*P* = 0.020, Table 2A). There was a trend toward increased SI in patients with CTS compared with healthy participants at the pisiform bone (*P* = 0.066), which reached significance at the level of the hamate bone (*P* < 0.0001). Signal intensity and CSA of the ulnar nerve were comparable between groups at all levels (*P* > 0.201).

**TABLE 2:**
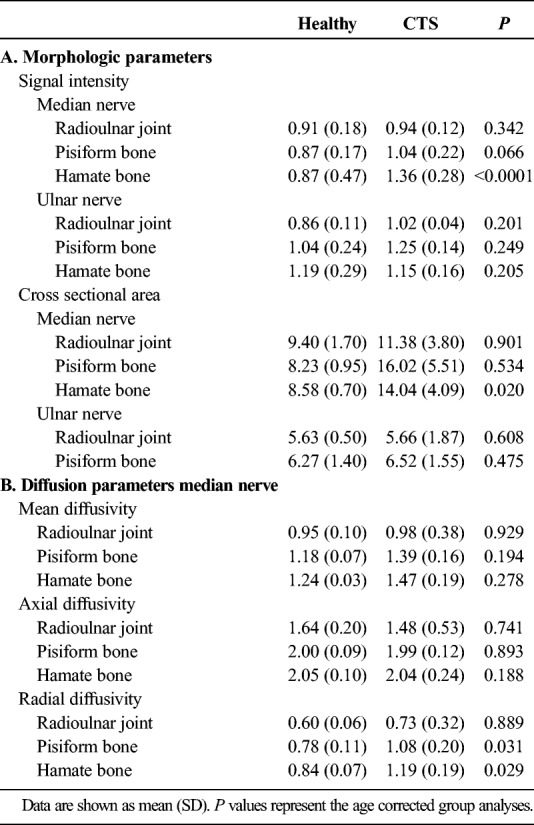
(A) Signal Intensity and Cross-sectional Area of Median and Ulnar Nerves in Patients with CTS and Healthy Controls and (B) Mean, Axial, and Radial Diffusivity Values of the Median Nerve

### DTI Images at 7 T Are Distorted But Can Be Corrected With TOPUP

**FIGURE 2:**
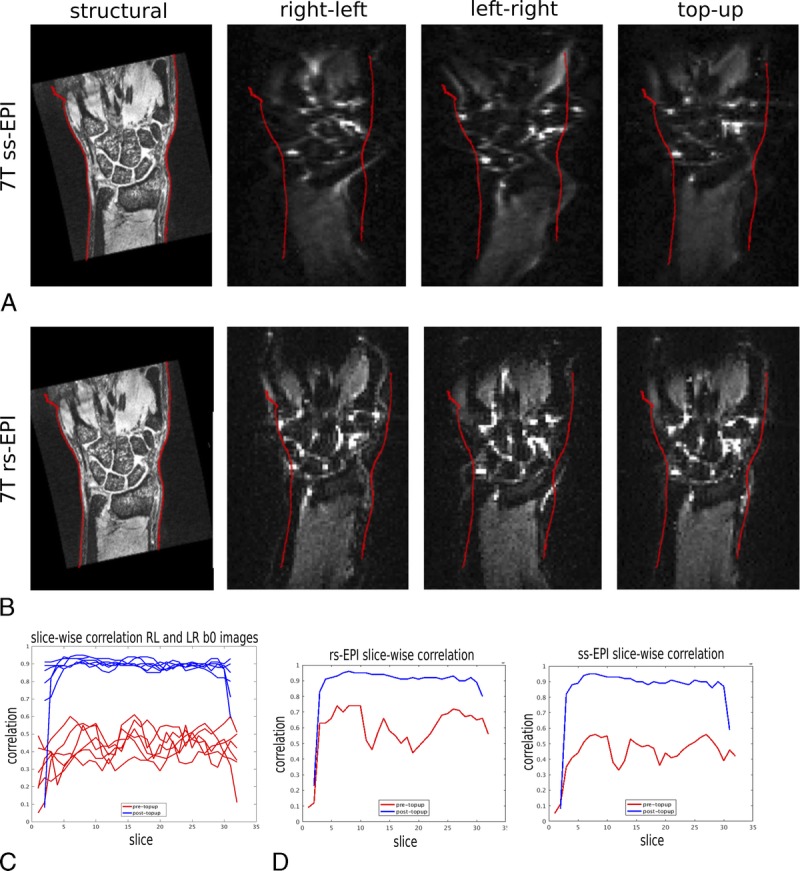
A and B, Diffusion imaging at 7 T induces significant distortion, which can be corrected by combining acquisitions with reversed EPI phase-encoding blips. A, Image shows frontal plane slices of morphologic, unweighted right-left phase encode blip, unweighted left-right phase encode blip, and the combination of unweighted opposing blips (TOPUP) for the ss-EPI acquisition. The red lines show the edges of the morphologic scan overlaid onto the diffusion images. There is extensive distortion, which can be substantially mitigated with TOPUP correction. B, Image shows the rs-EPI acquisition, which reduces distortions and achieves a more anatomically faithful image after TOPUP correction, in comparison with ss-EPI images. C and D, Slice-wise correlation between reversed phase-encode blips b = 0 acquisitions (RL, right-left; LR, left-right). C, Before TOPUP (red), correlations between reversed phase encode acquisitions are low, indicating substantial distortions at 7 T. TOPUP (blue) successfully corrects the distortions and increases the correlations. D, Correlations between uncorrected blip reversed acquisitions (red) within the same scan session were higher for rs-EPI compared with ss-EPI sequences, but could largely be corrected with TOPUP (blue).

Distortion artifacts were pronounced, especially at bone-bone interfaces at the level of the carpal bones (Fig. 2, A and B). This was apparent by low correlations of reversed phase-encoded acquisitions (mean *r* = 0.44 [SD 0.05], Fig. 2C). TOPUP achieved substantial distortion correction (Fig. 2, A and B; mean *r* = 0.90 [SD 0.01] after TOPUP; *P* < 0.0001; Fig. 2C). EDDY correction was also found to be crucial in aligning the diffusion-weighted images, which are distorted differentially as a result of changing the diffusion gradient directions (Appendix Figure 2, Supplemental Digital Content, http://links.lww.com/RLI/A392).

The rs-EPI images provided reduced image distortion compared with ss-EPI images as demonstrated by a direct comparison in the same participant during the same session (mean correlation rs-EPI *r* = 0.62 vs ss-EPI *r* = 0.47; Fig. 2, A and B). After TOPUP correction, the slice-wise correlation was comparable between ss-EPI and rs-EPI (*r* = 0.91 vs 0.92, Fig. 2D).

Although SNR was comparable between ss-EPI (5.36) and rs-EPI acquisitions (4.07), SNR efficiency was a factor 2 lower than rs-EPI acquisitions (1.01 vs 2.24 in ss-EPI) due to longer scan time per image. This lower SNR efficiency offset the benefits of reduced artifacts in the rs-EPI data.

### Registration of DTI to Morphologic Scans Is Successful at 7 T

**FIGURE 3:**
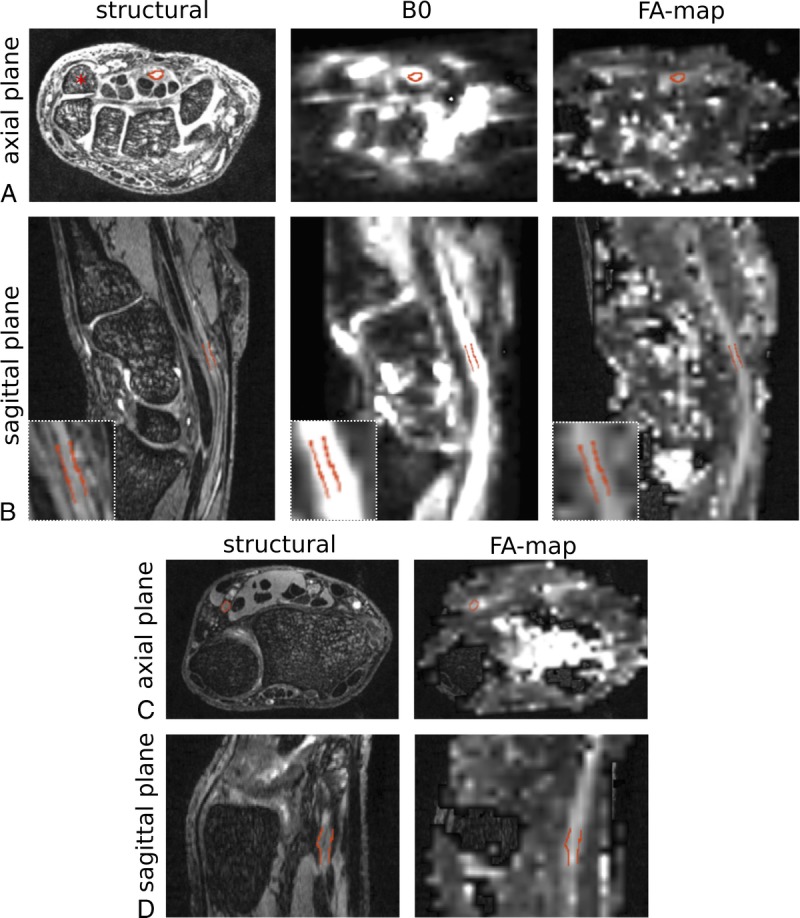
A and B, Registration of the unweighted (b = 0) images and fractional anisotropy (FA) maps to the morphologic images was successful at 7 T in the transaxial plane (A), which is commonly used for diagnostic purposes, as well as sagittal planes (B). The median nerve is delineated in red on the morphologic images at the level of the pisiform bone (asterisk). Registration reveals good alignment with the hyperintense median nerve structure on the unweighted b = 0 images. The median nerve is also clearly indicated by a region of elevated signal in the FA maps, with accurate registration to the morphologic image. Insets show magnifications of the outlined nerve. Because of the left-right phase encode direction, distortion artifacts are more apparent in the transaxial images (A) compared with the sagittal images (B). However, registration remains accurate. C and D, Fractional anisotropy (FA) maps of the ulnar nerve at the wrist are successfully depicted. Transaxial (C) and sagittal (D) sections of the wrist demonstrating successful registration of the ulnar nerve (red outline) on the T2-weighted image (left) with the FA map (right).

Despite the significant distortion artifacts in 7 T EPI data, FMRIB's Linear Image Registration Tool was capable of achieving an accurate registration of the distortion-corrected diffusion images with the morphologic images for both the median (Fig. 3, A and B) and ulnar nerve (Fig. 3, C and D). This was apparent by perfect alignment of the median nerve in morphologic scans and FA maps in 12 participants and partial alignment in 2 participants.

### Fractional Anisotropy Maps Clearly Delineate the Peripheral Nerves at 7 T

**FIGURE 4:**
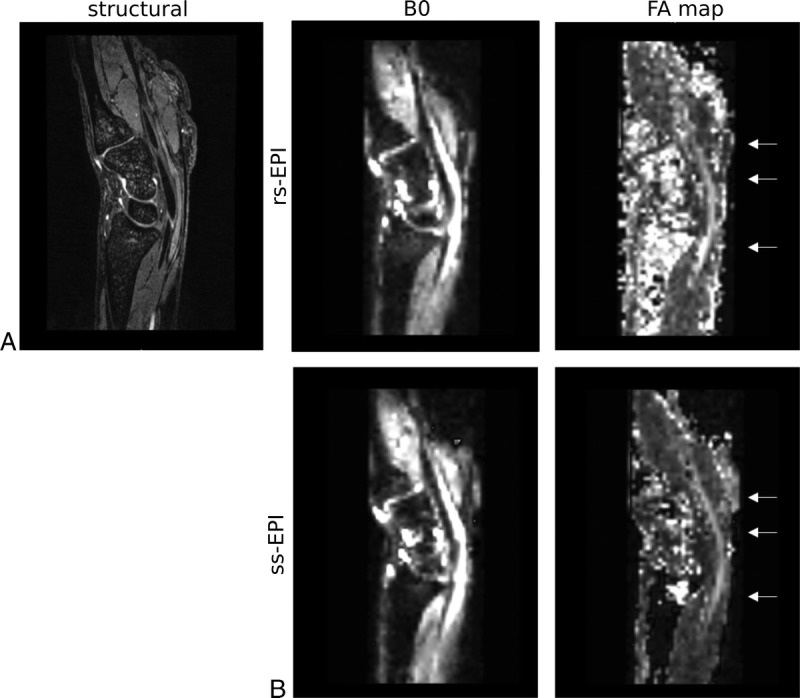
A and B, The median nerve is clearly delineated in fractional anisotropy (FA) maps at 7 T. A, Image shows sagittal slices of morphologic, unweighted b = 0, and FA maps at 7 T acquired with rs-EPI sequences and (B) ss-EPI sequences. The extraneural variability in the FA values is higher with the rs-EPI compared with ss-EPI sequences. The arrows depict the level of the radioulnar joint, pisiform bone, and hook of hamate, where FA within regions of interest of the median nerve were compared.

On FA maps, the median nerve could be distinguished from surrounding structures as a hyperintense line (Fig. 4, A and B). Measuring FA at the 3 wrist levels revealed comparable values across rs- and ss-EPI sequences (ss-EPI pisiform, 0.55; rs-EPI, 0.59). In accordance with the lower SNR efficiency, standard deviations within ROIs were consistently higher in rs-EPI (0.032) compared with ss-EPI acquisitions (0.019). In addition to the lower FA variability, the median nerve was more clearly separated from its surrounding structures in the ss-EPI acquisitions (Fig. 4, A and B).

**FIGURE 5:**
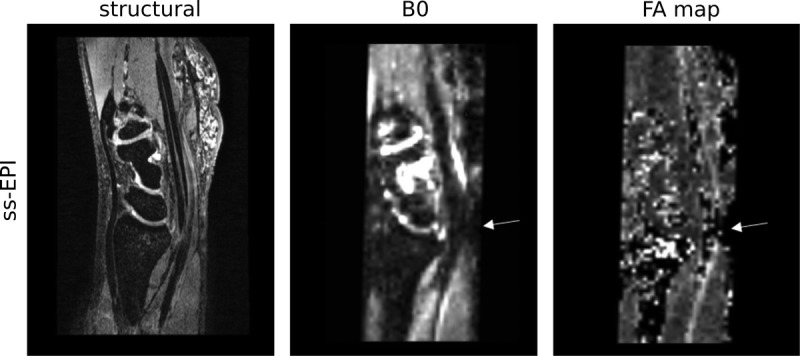
Although the distortions at 7 T could be effectively corrected in most participants, the bone-bone interfaces at the level of the carpal bones induced significant distortions in some participants. These severe distortions could not be corrected by TOPUP, leading to focal signal loss in TOPUP-processed ss-EPI images and subsequent FA map reconstruction (arrows, sagittal slices).

Although most distortions could be effectively corrected, severe distortions caused focal signal loss, which could not be corrected in some participants (n = 1/6 healthy, n = 3/8 patients, Fig. 5). However, this signal loss remained localized to the radioulnar joint.

### 7 T Imaging Detects Altered Diffusion Parameters in Patients With CTS

Intertester reliability was good to excellent for all DTI parameters (ICC, 3.2 [95% confidence intervals]: FA, 0.955 [0.860–0.986]; MD, 0.867 [0.586–0.957]; raD, 0.885 [0.641–0.963]; axD, 0.919 [0.721–0.971]; *P* < 0.0001).

**FIGURE 6:**
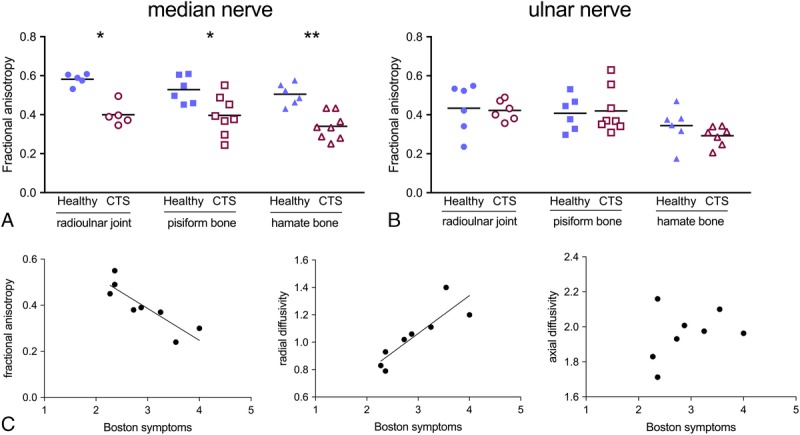
A, Median nerve fractional anisotropy (FA) at 7 T is lower in patients with CTS (open red symbols) than healthy participants (blue filled symbols). FA of the median nerve is shown at the level of the radioulnar joint (*P* = 0.016), the pisiform (*P* = 0.047), and the hamate bone (*P* = 0.001). B, FA of the ulnar nerve is comparable at the different wrist levels between patients with CTS (red open symbols) and healthy controls (blue filled symbols, all *P* > 0.248). C, Fractional anisotropy (FA) negatively correlates with symptom severity as measured with the Boston symptom questionnaire (*P* = 0.005). Radial diffusivity positively correlates with symptom severity in patients with CTS (*P* = 0.005). No correlations of axial diffusivity with patients’ symptoms was apparent (*P* = 0.413).

There was a significant decrease in FA of the median nerve at all wrist levels in patients with CTS compared with healthy controls (*P* < 0.047; Fig. 6A). The ulnar nerve, which is not directly affected in CTS, demonstrated comparable FA (*P* > 0.248, Fig. 6B). Although MD and AD were comparable between groups (*P* > 0.188), RD was significantly higher at the pisiform and hamate bone in patients with CTS (*P* < 0.031, Table 2B).

### Diffusion Values Correlate With Patients' Symptoms

Fractional anisotropy and RD (*r* = −0.866 and 0.866, respectively, *P* = 0.005), but not AD (*P* = 0.413) strongly correlated with symptom severity (Fig. 6C). None of the diffusion (*P* > 0.177) or morphologic parameters (*P* > 0.020) correlated with electrodiagnostic test parameters after Bonferroni correction. Electrodiagnostic test parameters did not correlate with symptom severity (*P* > 0.365).

## DISCUSSION

Our data demonstrate the feasibility and challenges of morphologic and diffusion peripheral nerve imaging at 7 T. In our data, diffusion but not morphologic parameters correlate with symptom severity in patients with CTS.

Anatomical imaging of peripheral nerves has been previously attempted at 7 T.^4–6,25^ Our SSFP sequence with T2/T1 contrast allowed us to achieve an approximately 4 times smaller voxel volume (0.2 × 0.2mm, 0.4-mm slice thickness) than previously reported for pure T2-weighted sequences at 7 T (eg, 0.39 × 0.36mm, 0.4-mm slice thickness).^4^ At this high resolution, it was possible to visualize single fascicles within the median nerve and small branches of peripheral nerves (eg, superficial radial nerve) as well as other structures within the carpal tunnel such as the flexor tendons and their sheaths. Such highly resolved images might be of diagnostic value for neuropathies affecting inaccessible small diameter nerve trunks (eg, radiculopathy, thoracic outlet syndrome). Although SSFP sequences reveal fine anatomical details of different structures within relatively short scan times, they are not specifically optimized for neural tissues such as classical MR neurography sequences (eg, spin echo acquisitions with fat saturation). Future work at 7 T could explore the feasibility of increasing SSFP T2 contrast with T2 preparation pulses^26^ and the addition of fat suppression^27^ to optimize neural contrast.

The identified increased CSA and SI at the level of the hamate bone in patients with CTS are in agreement with data collected at lower field strength.^28^ The nonsignificant trend at more proximal wrist levels is most likely attributed to the small sample of this technical development.

Although DTI of peripheral nerves has been performed at lower field strength (eg,^29–32^) and has been suggested to outperform the diagnostic accuracy of morphologic imaging alone,^32^ this is the first 7 T DTI study in a patient population. As expected, the 7 T acquisitions come with several challenges. First, distortion artifacts worsen with increasing field strength. We were able to successfully correct the majority of distortion artifacts caused by B0 inhomogeneity and eddy-currents. Distortion artifacts caused by tissue-specific parameters (eg, susceptibility differences, chemical shift) may have led to exacerbated distortions at the level of the carpal tunnel, where the anatomy is highly complex. Unfortunately, these led to uncorrectable signal loss in the FA maps in a minority of participants. Signal loss was not encountered more proximal or distal of the tunnel, suggesting that EPI-based peripheral nerve imaging at 7 T is more reliable in less anatomically complex regions.

Another challenge of DTI is the quality of the EPI images (low spatial resolution and artifacts), which can make it difficult to confidently outline specific structures on diffusion maps. The successful registration of morphologic and diffusion images achieved here will ensure correct structure identification. Because of some remaining blurring in the phase encode direction, the peripheral nerves are slightly wider on the axial diffusion images compared with the morphologic images. In the sagittal plane, however, where distortion artifacts are much less pronounced, the peripheral nerves in the morphologic images and FA maps almost perfectly aligned. To minimize partial volume effects, we recommend outlining an ROI within the peripheral nerves in FA maps, then confirming a correct alignment in the morphologic images.

Long scan durations are a further challenge in DTI, which can be exacerbated at 7 T due to the higher specific absorption rate (SAR). The use of a local radiofrequency transmit coil permitting higher SAR limits enabled us to develop protocols with an acceptable scan duration for clinical use. The advantages of the ss-EPI sequences (higher SNR efficiency leading to better delineation of the nerves in FA maps in a shorter scan time) outweighed the reduced distortions in rs-EPI images.

Recent developments such as simultaneous multislice imaging^15,16,33^ and a reduced number of readout segments with partial Fourier reconstruction^34^ have previously been used to accelerate rs-EPI of peripheral nerves and the brain at 3 T and have recently been optimized for the brain at 7 T with low-SAR PINS RF pulses.^35^ These methods could be used in future studies to increase the SNR efficiency of rs-EPI and shorten the scan times.

We found a reduction in FA of the median but not ulnar nerve in patients with CTS. In addition, median RD increased but AD did not change. The pattern identified here is similar to recent DTI studies at lower field strength in patients with CTS^24,36,37^ or other peripheral neuropathies.^31,38,39^ Reduced FA and increased RD are also the predominant findings in experimental nerve injury models, where they correlate with histological markers of axon and myelin degeneration,^40–42^ or the presence of inflammation-induced edema.^43^ In humans, correlation of diffusion parameters with electrodiagnostic parameters thought to reflect axonal damage or demyelination reveal conflicting outcomes.^24,44,45^ This may be attributed to the limited ability of electrodiagnostic tests to differentiate axonal from myelin damage^46,47^ as well as their inability to determine changes in small fibres (C and Aδ), which are affected early in CTS.^10^ Importantly, intraneural edema, which is likely present in entrapment neuropathies such as CTS^48^ and can influence DTI parameters,^43^ cannot be depicted with electrodiagnostic tests. Here, MRI parameters did not correlate with electrodiagnostic measures. However, FA and RD strongly correlated with symptom severity. Peripheral nerve FA has previously been found to correlate with symptoms in patients with CTS^49^ and lumbar radiculopathy.^39^ Those results and our findings suggest that FA and RD are imaging correlates for symptom severity in patients with entrapment neuropathies. This is of specific relevance in CTS, where the diagnostic criterion standard (electrodiagnostic testing) shows no or at best only a modest correlation with patients' symptoms.^50^

Although our main outcome measures were significant due to the large effect sizes, the findings in this preliminary report are based on a relatively small proof-of concept sample. Future work will have to determine optimal diagnostic cutoff values of DTI parameters as previously reported at lower field strength.^24^ Importantly, the here reported diffusion and morphologic parameters at 7 T in healthy participants and patients with CTS will provide important baseline measurements for future studies in larger patient populations of different etiologies.

In conclusion, our data demonstrate the feasibility of morphologic and diffusion peripheral nerve imaging at 7 T field strength. Diffusion tensor imaging at 7 T suffers from EPI artifacts, which are largely correctable in postprocessing. The strong correlation with the Boston symptom scale suggests a role for FA and RD as imaging correlates for symptom severity.

## Supplementary Material

SUPPLEMENTARY MATERIAL
