# Supplementary figures and images for: Feasibility of Diffusion Tensor and Morphologic Imaging of Peripheral Nerves at Ultra-High Field Strength
Source: Invest Radiol. 2018 Nov 7;53(12):705–13. doi: 10.1097/RLI.0000000000000492 (PMC6221405; doi:10.1097/RLI.0000000000000492)

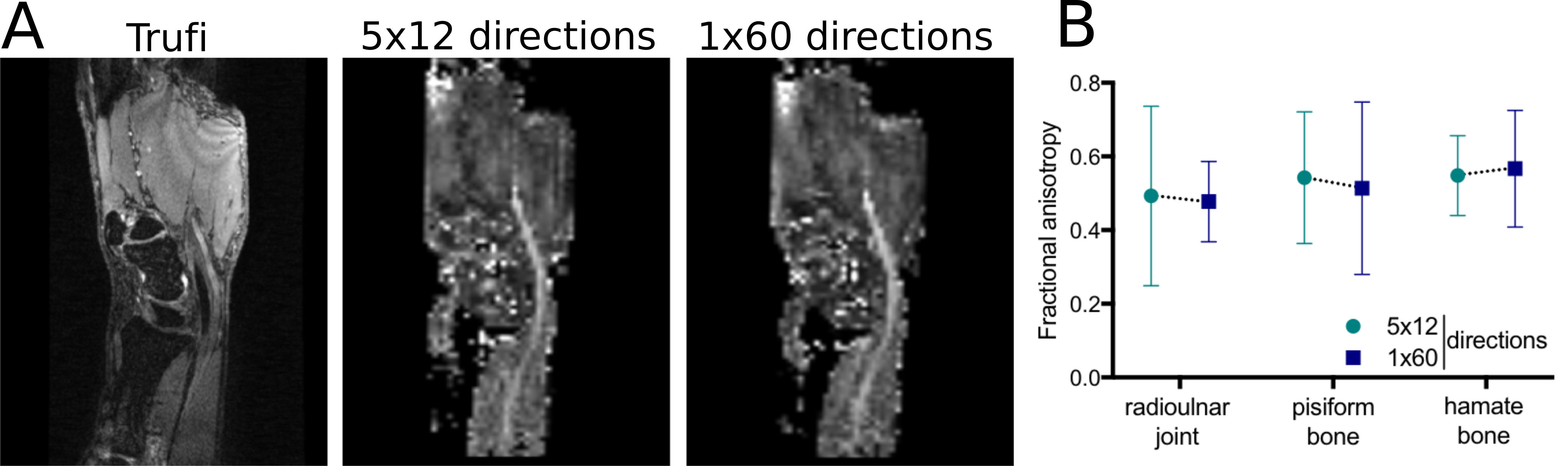

Supplement: SUPPLEMENTARY MATERIAL [file rli-53-705-s002.tiff]

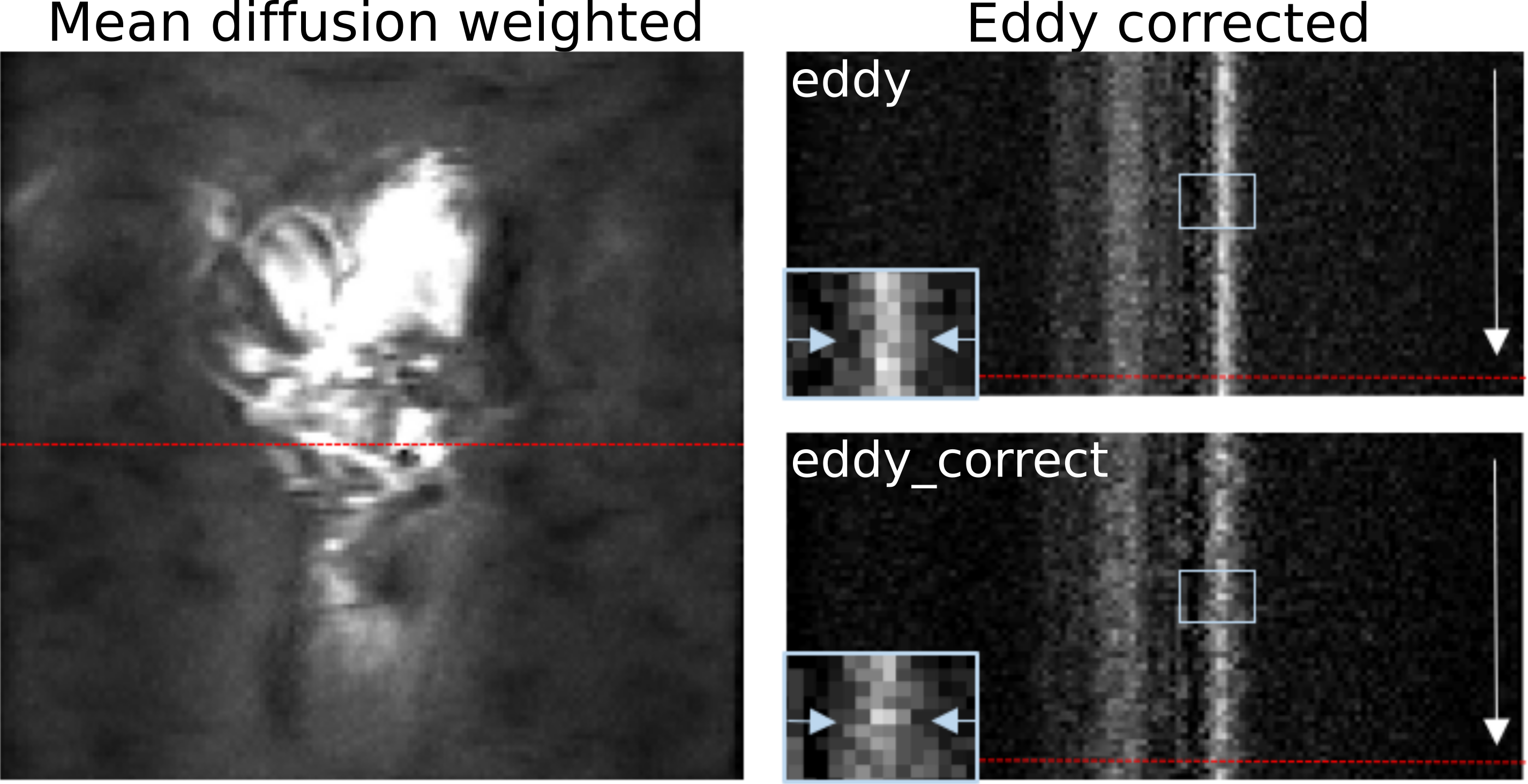

Supplement: SUPPLEMENTARY MATERIAL [file rli-53-705-s003.tiff]
